# Fermentation: Its Cause and Effects

**Published:** 1890-01

**Authors:** Ernest Laplace

**Affiliations:** A.M., M.D. (Paris), Professor of Pathology in the Medico-Chirurgical College, Philadelphia


### International Dental Journal.

Vol. XI. January, 1890. No. 1.

### Original Communications.[1]

### FERMENTATION: ITS CAUSE AND EFFECTS.[2]

BY ERNEST LAPLACE, A.M., M.D. (PARIS), PROFESSOR OF PATHOLOGY IN THE MEDICO-CHIRURGICAL COLLEGE, PHILADELPHIA.

Gentlemen,—All that lives must die; and dying, disintegrate and resolve itself into elements that enter into new cycles of usefulness. This phenomenon of universal disintegration must have aroused the interest of the human intellect from the first moment that it reached such a state of perfection as led it to inquire into the causes of things. If this disintegration did not exist, the matter of organized beings would encumber the earth, and the law of perpetuity of life would be compromised. A great phenomenon presides over this work; this phenomenon is fermentation.

Our forefathers may have been savages; but they were clever and observant ones. After organizing their own rude arts, they turned beasts into servants; they founded agriculture; planted the grape. This fruit was surely antediluvian, for we are told that Noah, on leaving the ark, planted a vineyard, drank of the wine,

[1] The editor and publishers are not responsible for the views of authors of papers published in this department, nor for any claim to novelty, or otherwise, that may be made by them. No papers will be received for this department that have appeared in any other journal published in this country. The journal is issued promptly on the 15th of the month.

[2] Read before the Odontological Society of Pennsylvania, at its regular meeting, November 2, 1889.

and experienced its consequences. But though wine and beer are as old as history, it was not until within the last few decades that anything positive has been known as to the true mode of their formation. Our knowledge in the matter has been almost identical with our knowledge of medicine,—that is empyrical. By this is meant that we had observed the facts, aside from the principles which produce them, and which are essential for a true understanding of them. In a word, when light dawned upon the true secret of the manufacture of beer, by the immortal discoveries of Pasteur, that same beam spread itself over the whole realm of medicine; was the light of regeneration to the noblest of sciences.

The brewer learned from long experience the conditions, not the reasons, of success. Often, however, the brewer's beer has fallen into rottenness without any accountable cause. It is the hidden enemies against which the physician, like the brewer, has had to contend that recent researches are dragging into the light of day, thus preparing the way for their final extermination.

While still a young man, Pasteur, who was then a professor of chemistry in the École Normale, of Paris, was attracted by a note of the German chemist Mitscherlich, in which he said to the Academy of Sciences: "The tartrate and paratartrate of soda and ammonia have the same chemical composition, the same crystalline form and angles, the same specific weight, and the same double refraction. Dissolved in water, the refraction is the same. But the dissolved tartrate turns the ray of polarized light to the left, whereas the paratartrate is *indifferent.* But," adds Mitscherlich, "the nature and the number of the atoms is identically the same." Pasteur, who was then but twenty-five years of age, discovered that the crystals which turned polarized light were not symmetrical. He noticed that all products of organic life were dissymmetrical, such as starch, quinine, strychnia, etc., while all mineral crystals, or products of the inorganic world, were symmetrical. He immediately suspected that tartrates were organic,—that is, connected directly or indirectly with life. It was a well-known fact that a German manufacturer of chemical products having thrown away some impure tartrate of lime, mixed with albuminoid materials, this had fermented, giving rise to different products. Pasteur reproduced the fermentation in the following way: Taking some tartaric acid, he added a certain amount of albuminous material, and placed it in an incubator. When fermentation had taken place, Pasteur found innumerable small living organisms, and after the process had stopped he polarized the liquid, and found that, whereas before fermentation the polarization was to the left, it had now turned to the right.

His suspicion was realized.

Hence, this sudden change of the direction of the ray of polarized light was accompanied by a great development of small living organisms during a process heretofore known simply as fermentation. This was the first hint ever gotten of the influence of a living micro-organism upon organic substances. This micro-organism was not present apparently when the mixture was placed in the incubator, and now it existed in swarms in the disintegrating mixture. They surely grew during the fermentation.

Up to this time the most incomplete notions had been entertained as to the true cause of fermentation. *Liebig* said "it was an acidification of albuminous substances when in contact with air;" *Gay-Lussac* thought that the oxygen of the air was the causative agent, for he had noticed that wine had turned sour from being poured from one vessel into another; Berzelius and Mitscherlich said that ferments acted by catalysis,—that is, by their presence; Schwann and Cagnard Latour noticed that a living rounded body was present in the manufacture of beer, but it did not occur to them to ascertain what part was played by this organism. As soon as the malt is mixed with hops it is boiled and allowed to cool; this infusion is called the wort, and that is placed in vessels with but one aperture open to the air. Here it is mixed with the yeast. Soon after a brown froth forms on the surface, which is really new yeast, and issues from the aperture falling like a cataract into troughs prepared to receive it. Whence is this new yeast? Weigh it before and after. The brewer sows ten pounds and he collects fifty pounds. Shall we say that this is spontaneous? Are we not reminded of the seed that has fallen in good soil and brought forth fruit fifty- or a hundred-fold? In fact, this seed can be seen budding under the microscope before our own eyes. It is a minute plant, the torula cerevisiæ. This marks a distinct epoch in the history of fermentation. But Liebig was loath to accept the growth of this plant as the cause of fermentation, and maintained that its life had nothing to do with the process, that it was a purely chemical one, and that it was the chemical nature of yeast, not the fact that it was alive and could develop life, which produced fermentation. In a memorable demonstration, Ludersdorf proved the error of Liebig's assertion, and that yeast acted as a ferment because of its organized or living character. He destroyed the cells of yeast by rubbing them on a ground glass plate, and he found that, with the destruction of the organism, the chemical nature remaining the same, the power to act as a ferment disappeared totally. No experiment could possibly be more conclusive.

But in the manufacture of wine no yeast is added. The grape is pressed, and the juice ferments after a short while. The torula soon make their appearance, however, and where do they come from? If the filtered grape-juice be boiled, so as to destroy the germs it contains, and be put in germless air, it will never ferment. All the material for spontaneous generation is there, but the life both in the grape-juice and the air being destroyed, no new life can be produced in the shape of fermentation.

Pasteur has pushed this demonstration still further. The grape is sealed by its own skin from contamination by the air. He contrived a way of extracting the juice without its touching any contaminated substance and placing it in pure air; it did not ferment; then taking the skin of the grape and brushing the delicate grayish dust upon this non-fermenting juice, fermentation soon developed, and the yeast-plant appeared in great abundance; proving that the grape carries its yeast upon its own self. For thousands of years, therefore, the wine-grower has done unconsciously what the brewer does purposely.

The germ of the yeast-plant exists in the air, but not in quantities sufficient to insure rapid fermentation, such as the brewer desires, hence the brewer puts in a quantity himself. Pasteur has defined fermentation as life without air. These germs live on oxygen, as we do, and give off carbonic acid gas; but they do not take their oxygen from the air, they take it from the substance upon which they grow; hence they do not need the oxygen of the air for their development. Hence fermenting substances are placed in vessels with but a small aperture to the open air, where the yeast imbibes oxygen and pours forth carbonic acid. Where does it get the oxygen? It is wrenched from the liquid upon which it grows; liberates carbonic acid gas, and leaves the liquid product as our familiar alcohol.

And in the same way exposing alcohol to the action of the ferment known as the mycoderma aceti, acetic acid will be the result.

The air is full of germs of ferments differing from the alcoholic leaven. Expose milk to the air, and coagulation will take place; small globules of butter appear,—the butyric acid fermentation. Within a short while larger organisms are seen wriggling in swarms through the preparation. In curdled milk are found other organisms linked together, as beads on a string,—that is the lactic acid fermentation. Examine putrefying milk, and it will be seen to swarm with millions of small and larger germs, showing wonderful alacrity of motion. Keep your milk from the influence of the atmosphere, or boil it so as to kill the germs within it, and it will remain sweet, the germs being destroyed. Expose meat to the atmosphere, and it will soon putrefy; it will swarm with the germs of putrefaction, and will soon stink. Keep the germs away, and it will not putrefy. Thus we begin to see that within the world of life, to which we ourselves belong, there is another world requiring the microscope for its discernment, but which, nevertheless, has a most important bearing upon our welfare.

Gathering these facts together, and analyzing them, we see that there are two elements always in action, a seed and a soil: the seeds are floating continually in the atmosphere about us; the soil is the particular substance upon which these germs fall, and at the expense of which they grow. It follows also that the greatest analogy exists between these various germs in the atmosphere and the seeds of various plants that may be wafted by the wind from one spot to another, and which develop when they happen to fall upon a suited soil.

Another fact is that, just as when you sow corn, corn is reaped and not barley, so each particular germ grows its particular kind. Supposing you take a handful of seeds of various flowers, and sow them in the same soil, then the different plants will grow alongside of one another. So also, if various sorts of germs have access to the same soil, they may grow plentifully together, as takes place in putrefaction, where germs of many kinds are seen growing and decomposing the soil upon which they grow, liberating not carbonic acid, as in fermentation, but sulphuretted hydrogen,—the foul smell of putrefaction.

All germs will not grow on the same soil, just as all seeds will not grow in the same ground; some plants being indigenous to some countries and others to different climates. But a very astonishing fact is that one germ, after developing in a particular soil, may leave that soil in such a state as will render it favorable to the development of a germ which could not have developed there before. Such is indeed the case with the mycoderma aceti, which could not have developed in the sweet solution. First, the yeast-plant developed there, changing the sugar into alcohol, and now the mycoderma aceti, falling into the alcohol, grows abundantly, changing this alcohol into acetic acid or vinegar.

The most important and practical portion of the whole knowledge of the nature and development of micro-organisms is the study of the changes incident to their growth in the soil upon which they develop. The yeast-plant left the sugar changed into alcohol, whose chemical nature and physiological effects are quite different from sugar. The mycoderma aceti has changed the alcohol into vinegar, whose chemical nature and physiological effects are vastly different from alcohol. Likewise the lactic acid germ has produced in milk, which was once sweet, a substance (lactic acid) having corrosive properties, and which curdles the milk.

This new product, which results from the decomposition incident to the development of a germ, is called in medicine a ptomaine. When germs are absorbed from the atmosphere and produce certain diseases, the albuminoids are decomposed within us, and this ptomaine or fermentative product is the chemical poison formed, which, being resorbed by the economy, produces those physiological symptoms characteristic of a disease.

And would a substance putrefy without the action of germs? In other words, is there such a thing as spontaneous generation? Tyndall's and Pasteur's admirable researches have set this question at rest. One will suffice. Having made veal broth, Pasteur placed it in a round vessel, with but a small aperture. This was raised to a temperature of 115° C. for half an hour, so as to destroy all the germs within it, and the tip end of the flask was soldered, so as to prevent further air from coming in contact with the broth. A number of flasks so treated were placed aside. I have one in my possession thus prepared, many years ago, by Pasteur himself, and its contents are as pure as on the day of its preparation. This shows conclusively that by heat he had sterilized the liquid and interior of the flask, and, having soldered the end of the flask, thus preventing any germs of the atmosphere from having access to the fluid, there was no possibility of life developing in it; hence it remains pure, and is likely to remain pure indefinitely.

Now, this simple experiment was a master stroke of genius, for on it is founded our whole system of modern pathology and hygiene.

Do we wish to stop fermentation, putrefaction, contagious and epidemic diseases, we must repeat Pasteur's experiment,—sterilize first; then prevent the germs from having further access to the parts thus purified.

Strange to say, this process of purification was applied to the canning of goods before it was practically applied to scientific purposes. In fact, canned fruits of all sorts are prepared exactly after the manner of Pasteur's broth experiment. They are raised to a high temperature and kept in air-tight vessels. And what occurs when canned goods become spoiled? Simply, the germs of the air have gotten into the goods, either through some small aperture in the vessel or sufficient heat was not applied at first to destroy them.

Lister, in England, was the first to make a practical application of this to suppuration in wounds. He saw the analogy between the foul smell of a suppurating wound and the process of putrefaction, and concluded that, should he succeed in destroying the germs which had started this putrefying process, and should he prevent further germs from having access to the wound, this putrefaction would cease and the wound heal kindly without suppuration. This he did by sterilizing the wound. There are two ways of sterilizing: (1) by heat; (2) by chemical agents: carbolic acid, sublimate, etc., which have the property of destroying the vitality of most micro-organisms; and when used in proper strength, do so without impairing the tissues with which they come in contact. The wound is then covered with several layers of cotton that has been sterilized or purified by heat,—and this prevents the germs of the air from having further access to the wound; for as these germs fall upon the outer layers of the cotton, they are caught by the meshes of the small cotton fibres and are not allowed to get any nearer the wound; during this time the normal and unimpeded process of repair goes on, and healing takes place without suppuration or putrefaction produced by germs. And by this glorious discovery Lister has blessed humanity with a reduction in mortality from major surgical operations; a mortality reduced from fifty per cent. before the days of antisepsis to two or three per cent. at the present day in the best-conducted hospitals. Besides, it has widened the domain of surgery, bringing to the surgeon success in such operations, in which he could not hope for success should suppuration take place.

Applying these same principles to dental surgery which is a special branch of general surgery, we are struck by the frequency of processes of putrefaction or fermentation in the mouth. And why? Because the germs of the atmosphere, which we constantly breathe through the mouth, lodge upon some remnant of food buried in the sulci, fissures, and proximal surfaces of the teeth, and find there a suited soil, accompanied by heat and moisture; these germs develop and cause putrefaction, as evidenced by the foul odors from the mouth. Besides putrefactive processes, a fermentative process also may take place in the presence of sugar which produces a corrosive element that destroys the enamel. The germs sink into this impaired spot, and the same deleterious agent being generated there, the process of decay attacks the dentine, the pulp, and an extensive cavity follows. Such being the case for a healthy tooth, well paved with enamel, the process is a much easier and more rapid one when through some accident a mechanical abrasion already exists.

To the honor and credit of Professor W. D. Miller, of Berlin, be it said that he was the first to discover that the corrosive substance so deleterious to enamel, and which results from fermentation in the mouth, was lactic acid, which enters into composition with the calcium salts of the tooth, producing a lactate of lime.

This being the case, as a student of pathology and practitioner in surgery, I conjure you to apply to your special branch of surgery the principles of antisepsis that Lister has applied to general surgery, vouching that you will meet the same grand success. To reach this end, sterilize, and prevent the further access of germs to the parts. Sterilize with the acid sublimate solution, destroying the organisms at one sitting. But when the pulp is dead use the heated platinum broach in the root-canals, then use the antiseptic solution, which will penetrate the minute nooks of the cavity, and destroy those few germs that have perhaps escaped the heat; finally, plug the tooth with aseptic or antiseptic filling. And, gentlemen, as a physician, knowing how many general disorders, gastric and others, are caused by germs that found originally a brooding-place in the mouth, I implore you to give this question of oral disinfection your most scrupulous attention, feeling confident that a rational and persistent use of the antiseptic solutions now within our reach will afford you the fullest satisfaction, for in your treatment you will meet the same glorious success as is achieved in surgery as practised at your doors.

And now, having reviewed the various phenomena of fermentation, we see that in the eternal laws of the universe fermentation was destined as a power for good, and, like electricity and steam, it vastly benefits our existence, if only rightly understood and maintained within its proper bounds; that most of the harm to humanity resulting from fermentative processes is due to our still incomplete mastery of its laws; and that with the present strides of science, we will completely overcome those micro-organisms or fermentative agents that are deleterious to man and his surroundings.

Such is the triumph of the scientific age in which we live, that has disclosed a world about us which we knew not of; a world of beings consisting of enemies as well as benefactors, in constant and intimate relation with each of us.

We know of numberless stars above, infinitely large, but this newly-discovered world of infinitely small beings, its laws and purposes, is to me not a lesser index to that Power, the Author of them all,—

> "That God which ever lives and loves,
> One God, one law, one element,
> And one far off divine event
> To which the whole creation moves."

